# Neurological symptoms in COVID-19: a cross-sectional monocentric study of hospitalized patients

**DOI:** 10.1186/s42466-021-00116-1

**Published:** 2021-03-12

**Authors:** Ummehan Ermis, Marcus Immanuel Rust, Julia Bungenberg, Ana Costa, Michael Dreher, Paul Balfanz, Gernot Marx, Martin Wiesmann, Kathrin Reetz, Simone C. Tauber, Jörg B. Schulz

**Affiliations:** 1 Department of Neurology, RWTH University Hospital, Aachen, Germany; 2 Department of Pneumonology and Internal Intensive Care Medicine, RWTH University Hospital, Aachen, Germany; 3 Department of Cardiology, Angiology and Internal Intensive Care Medicine, RWTH University Hospital, Aachen, Germany; 4 Department of Operative Intensive und Intermediate Care Medicine, RWTH University Hospital, Aachen, Germany; 5 Department of Diagnostic and Interventional Neuroradiology, RWTH University Hospital, Aachen, Germany; 6 JARA-BRAIN Institute Molecular Neuroscience and Neuroimaging, Forschungszentrum Jülich GmbH and RWTH University Hospital, Aachen, Germany

**Keywords:** SARS-CoV-2, COVID-19, Neurological symptoms, Neuro-invasive potential, Cognitive impairment

## Abstract

**Background:**

The SARS-Coronavirus-2 (SARS-CoV-2) invades the respiratory system, causing acute and sometimes severe pulmonary symptoms, but turned out to also act multisystematically with substantial impact on the brain. A growing number of studies suggests a diverse spectrum of neurological manifestations. To investigate the spectrum of symptoms, we here describe the neurological manifestations and complications of patients with proven SARS-CoV-2 infection who have been hospitalized at the RWTH University Hospital Aachen, Germany.

**Methods:**

Between March and September 2020, we evaluated common symptoms, clinical characteristics, laboratory (including cerebrospinal fluid (CSF) analysis), radiological, and electroencephalography (EEG) data from 53 patients admitted with a positive SARS-CoV-2 polymerase chain reaction (PCR). We used the Montreal Cognitive Assessment Test (MoCA) to screen for cognitive impairment, when feasible. We compared critically ill and non-critically ill patients categorized according to the presence of Acute Respiratory Distress Syndrome (ARDS).

**Results:**

Major clinical neurological features of hospitalized COVID-19 patients were coordination deficits (74%), cognitive impairment (61.5%), paresis (47%), abnormal reflex status (45%), sensory abnormalities (45%), general muscle weakness and pain (32%), hyposmia (26%), and headache (21%). Patients with ARDS were more severely affected than non-ADRS patients. 29.6% of patients with ARDS presented with subarachnoid bleedings, and 11.1% showed ischemic stroke associated with SARS-CoV-2 infection. Cognitive deficits mainly affected executive functions, attention, language, and delayed memory recall. We obtained cerebrospinal fluid (CSF) by lumbar puncture in nine of the 53 patients, none of which had a positive SARS-CoV-2 PCR.

**Conclusions:**

In line with previous findings, our results provide evidence for a range of SARS-CoV-2-associated neurological manifestations. 26% of patients reported hyposmia, emphasizing the neuro-invasive potential of SARS-CoV-2, which can enter the olfactory bulb. It can therefore be speculated that neurological manifestations may be caused by direct invasion of the virus in the CNS; however, PCR did not reveal positive intrathecal SARS-CoV-2. Therefore, we hypothesize it is more likely that the para-infectious severe pro-inflammatory impact of COVID-19 is responsible for the neurological deficits including cognitive impairment. Future studies with comprehensive longitudinal assessment of neurological deficits are required to determine potential long-term complications of COVID-19.

**Supplementary Information:**

The online version contains supplementary material available at 10.1186/s42466-021-00116-1.

## Background

In December 2019, a high number of patients infected with severe acute respiratory syndrome coronavirus 2 (SARS-CoV-2) was first reported in Wuhan, China and marked the beginning of a pandemic of unknown dimension in the twenty-first century. Although Corona Virus Disease 2019 (COVID-19) primarily affects the pulmonary system, neurological pathological manifestations in patients infected with SARS-CoV-2 have been reported already in early stages of the pandemic [1]. The growing number of studies on SARS-CoV-2-induced central nervous system (CNS) effects revealed a multitude of neurological implications ranging from meningitis, encephalitis, vasculitis, acute disseminated encephalomyelitis, neuropathies to SARS-CoV-2-associated strokes [2]. Several single-case reports of rapidly developed parkinsonism after SARS-CoV-2 infection gave rise to the idea that SARS-CoV-2 could also accelerate or aggravate pre-existing neurodegenerative disorders, or even induce them de novo [3, 4].

SARS-CoV-2, similar to the related corona viruses SARS-Coronavirus-1 (SARS-CoV-1) and the Middle East respiratory syndrome-related coronavirus (MERS), is potentially neurotrophic, and several mechanisms of CNS and peripheral nervous system (PNS) invasion have been proposed. The detection of viral particles and genomic sequences of SARS-CoV-1 in lymphocytes and monocytes implicates a hematogenous way as a possible mechanism of viral entry into the CNS [5, 6]. Another way of CNS invasion might be SARS-CoV-2 interaction with Angiotensin-converting enzyme 2 (ACE2) receptors which are not only expressed in lung and intestinal epithelium but are also found in the endothelial cells of the blood-brain barrier (BBB) [7]. Noteworthy, SARS-CoV-2 – like Influenza virus – can enter the brain via the olfactory nerve and induce an inflammatory status with subsequent immune reactions that affect surrounding brain regions as well [8]. In addition to the effects induced by the virus itself, other mechanisms including indirect effects of SARS-CoV-2 infection such as massive cytokine release, hypercoagulopathy and the organ damage it triggers, or sepsis itself have also been described as possible mechanisms responsible for sequelae [2].

One of the first local outbreaks of SARS-CoV-2 in Germany was registered in the neighboring districts of Heinsberg, leading to a high number of patients with COVID-19 transferred to the RWTH University Hospital Aachen. The neurological complications of COVID-19 patients that have been reported with increasing frequency are extremely diverse [9]. Therefore, we aimed a systematically investigation of neurological manifestations of COVID-19 patients treated at the RWTH University Hospital Aachen during the first pandemic wave. Our aim was to better understand the spectrum of neurological symptom manifestation in the context of their global occurrence.

## Methods

We present data from a single-center prospective study of 53 in-house COVID-19 patients hospitalized at the RWTH University Hospital Aachen in Germany, which were referred to the Department of Neurology between March and September 2020. From March 2020 on, we tried to systematically examine all admitted COVID-19 patients. However, in-depth neurological characterization was only possible in 53 of these 138 patients. There were several reasons that made it impossible to recruit more patients: (i) Initially, COVID-19 was considered a disease mainly affecting the lung, but this view was challenged soon. (ii) As soon as patients were diagnosed with COVID-19, they were isolated in dedicated COVID-19 wards. Contacts were restricted as much as possible and particularly in the beginning of the patients’ stay, neurological consultation had not been deemed necessary. However, the frequent occurrence of neurological symptoms changed this view throughout the initial weeks. (iii) The University Hospital Aachen being reference center, COVID-19 patients were transferred but their critical status did not allow for in-depth neurological examination or neurological imaging. (iv) Of the patients treated in the Intensive Care Units (ICU), 44% died [10]. Patients included in this report were systematically examined for neurological impairment. Patients with proven SARS-CoV-2 infection were isolated in specialized general wards in the Departments of Cardiology and Pulmonology, respectively, as well as Intensive Care Units (ICU), and were examined by neurology consultants (UE or MR).

A confirmed case of COVID-19 was defined as a positive real-time reverse-transcriptase–polymerase-chain-reaction (RT-PCR) of sample material from either bronchoalveolar lavage of intubated patients (*n* = 28) or nasal swab (other patients, *n* = 25).

Patients were further categorized according to the presence of acute respiratory distress syndrome (ARDS). According to the Berlin definition, ARDS is classified as mild, moderate, or severe by the relation of arterial partial pressure of oxygen (PaO2) to fraction of inspired oxygen (FiO2) at a threshold of 300, 200, and 100 mmHg, respectively [11].

We collected data on medical history, comorbidities such as vascular risk factors, cardiological and neurological pre-existing conditions and medication, from the patients’ clinical records. To obtain a comprehensive picture of neurological comorbidities, a detailed history regarding central and peripheral neurological symptoms was further obtained for each patient.

Neurological examination of intubated and sedated patients included assessment of consciousness using the Glasgow coma scale (GCS), screening for orientation and delirium, neck stiffness, examination of cranial nerves by assessment of pupils for size, symmetry and reactivity to light, primary eye position, motor and sensible response, deep tendon reflexes and pathologic reflexes (Babinski sign). If during weaning phase, patients were able to follow commands, then motor (tone, signs of rigidity and spasticity), sensitivity and coordination tests were carried out. Patients treated on the general isolation wards received a more comprehensive neurological examination including mental status, cranial nerves, motor system, deep tendon reflexes, sensitivity, coordination testing, nystagmus, tremor, assessment of ataxia and gait. With alert, conscious and cooperative patients, we performed the Montreal Cognitive Assessment Test (MoCA) to screen for cognitive impairment [12].

If further diagnostic clarification was needed following the neurological examination, lumbar puncture and cerebrospinal fluid (CSF) analysis, cranial computed tomography scan (CT) or magnetic resonance imaging (MRI) scan and/or electroencephalography (EEG) were carried out. Written informed consent was waived upon of urgent medical indication, particularly for patients who underwent invasive mechanical ventilation on ICU. For conscious patients, informed consent was obtained (RWTH University Hospital Aachen’s ethics committee approval number: 148/20). CSF was examined for elevation of cell count, level of protein, glucose, lactate, blood-brain-barrier (BBB) function, oligoclonal bands and presence of SARS-CoV-2 RNA. In addition, routinely collected laboratory parameters were evaluated with particular interest on D-dimers, Interleukin-2 (Il-2), Interleukin-6 (Il-6), ferritin, tumor necrosis factor alpha (TNF-alpha) and neuron-specific enolase (NSE).

Structural and vascular brain imaging were conducted either on CT, 1.5-Tesla magnetic resonance imaging (MRI), or 3-Tesla MRI. For most cases T1-weighted spin-echo (partly with gadolinium-based contrast agent), diffusion-weighted imaging (DWI), apparent diffusion coefficient (ADC)-imaging, gradient-echo T2, T2* susceptibility-weighted imaging, 2D FLAIR, and Time-of-Flight-Angiography (TOF) were available.

Electroencephalography (EEG) was done using the Sigma Medizin-Technik/Neurowerk Center V10.0.0.10-system. EEGs were recorded over 20 min with scalp electrodes placed according to the International 10–20-system. Low-pass and high-pass filters were set to 70 Hz and 0.3 Hz, respectively. Intensive Care Unit (ICU)-patients were examined using an eleven electrode-system including frontal (F), temporal (T), parietal (P), occipital (O) and central (Cz) electrodes. Even-numbered electrodes refer to electrode placement on the right side of the head, whereas odd numbers refer to those on the left.

A “z” refers to an electrode placed on the midline sagittal plane of the skull, (Fz, Pz, Cz). F3, F4, T3, T4, P3, P4, O1, O2 and Cz. Mobile patients from general isolation wards were examined using further electrodes Fp1, Fp2 (pre-frontal), F3, F4, T1, T2, T3, T4, T5, T6, P3, P4, O1, O2, Fz, Pz and Cz. Patients were stimulated by verbal commands, eye opening, and if necessary, by sternal rub (For a chronological list of all investigated patients with COVID-19 see Additional file 1).

Continuous variables were expressed as either medians and interquartile ranges or as simple ranges as adequate. Categorical variables were summarized as frequencies and percentages, respectively. No imputation was made for missing data. Since the sample of patients in our study was not derived from random selection, all statistics are descriptive only. Statistical analyses were performed using SPSS IBM version 26 with a type-I-error (α) set to 0.05 as the statistical threshold for significance. Groups were compared using non-parametric approaches using Pearson Chi^2^, Fisher’s Exact test, or Mann-Whitney U-test, depending on the comparison, as indicated.

## Results

### General aspects

From March through September 2020, 53 patients with a positive SARS-CoV-2 PCR underwent a comprehensive neurological examination and diagnostic work-up. Table 1 shows the demographic and clinical characteristics of the cohort. The median age was 63 years (interquartile range (IQR) 54–73 years) and 40% (*n* = 21) were female. There was no significant difference in the sex distribution (male or female). 61.5% (*n* = 28) of the patients suffered from ARDS in varying degrees of severity and all were treated on an ICU. The remaining 25 patients without ARDS were treated on the general isolation ward without ICU monitoring. The COVID-19 patients with severe ARDS were on average 4.5 years older (median age 61.5 years (IQR 56–68 years) than those without ARDS (median age 66 years, IQR 51–77 years). Male sex was predominant in both patients’ groups (ARDS 64% and non-ARDS 56%). Of the 28 patients with ARDS, 16 patients were treated with continuous veno-venous hemodialysis (CVVHD) and ten patients met the need of extracorporeal membrane oxygenation (ECMO).

**Table 1:** Clinical characteristics of patients with COVID-19

	Total N (%)	With ARDS N (%)	Without ARDS N (%)	*p*-value
Number of patients	53	28	25	
Age: years (Median + IQR)	63 (54–73)	61.5 (56–68)	66 (51–77)	.417
Female sex	21 (39.6)	10 (35.7)	11 (44)	.584
Evidence of COVID-19				
PCR (respiratory samples)	53 (100)	28 (100)	25 (100)	
Chest radiograph / chest computed tomography	53 (100)	28 (100)	25 (100)	
Comorbidities				
Arterial hypertension	36 (67.9)	19 (67.9)	17 (68)	.612
Diabetes mellitus	15 (28.3)	5 (17.9)	10 (40)	.160
Dyslipidaemia	11 (20.8)	6 (21.4)	5 (20)	.585
Smoking	5 (9.4)	3 (10.7)	2 (8)	.414
Ex-smoking	1 (1.9)	1 (3.6)	0	–
History of stroke/TIA	4 (7.5)	2 (7.1)	2 (8)	.509
History of traumatic brain injury	3 (6.7)	2 (7.1)	1 (4)	.543
Depression	7 (13.2)	5 (17.9)	2 (8)	.426
Coronary heart desease	12 (22.6)	8 (28.6)	4 (16)	.076
Atrial fibrillation	11 (20.8)	7 (25)	4 (16)	.509
History of myocardial infarction	8 (15.1)	8 (28.6)	0	.**004**
Carotid artery stenosis	2 (3.8)	1 (3.6)	1 (4)	.726
History of cardiovascular surgery (stent/bypass)	4 (7.5)	3 (10.7)	1 (4)	.533
Medication				
Antihypertensive drug	36 (67.9)	16 (57.1)	20 (80)	.562
Anticoagulant	21 (39.6)	18 (64.3)	3 (12)	**.0001**
Antidepressant	3 (6.7)	3 (10.7)	0	.335
Anticonvulsant	2 (3.8)	0	2 (8)	.466
Anti-psychotic	5 (9.4)	5 (17.9)	0	.101
Anti-parkinson	2 (3.8)	1 (3.6)	1 (4)	.934
Statins	6 (11.3)	2 (7.1)	4 (16)	.309
Immunosuppressant	8 (15.1)	5 (17.9)	3 (12)	.513
Other (Ibuprofen, Tilidine, L-Thyroxine, Pantoprazole, Salbutamol)	16 (30.2)	11 (39.3)	5 (20)	.126

*ARDS* Acute respiratory distress syndrome

Hypertension, atrial fibrillation and especially cardiovascular disease, including a history of myocardial infarction or cardiovascular surgery, were significantly more frequent among patients with ARDS than among those without ARDS. All eight patients with a history of myocardial infarction suffered from ARDS (X^2^ (1, *N* = 53) = 8.413, *p* < 0.05).

Only a few patients (*n* = 5) had a current or former cigarette smoking history. Patients who developed an ARDS had a significantly higher frequency of oral anticoagulation prior to hospitalization (X^2^ (1, N = 53) = 15.0928, *p* < 0.001). There was no statistical difference for any other group of medication. However, the proportion of patients taking antihypertensive drugs, such as angiotensin-converting-enzyme inhibitors (ACE inhibitors), calcium channel blockers (CCB) and diuretics, and statin therapy was higher in the non-ARDS group without reaching statistical significance.

### General and neurological symptoms

Fever and dyspnea were most prevalent at disease manifestation as initial general symptoms (Table 2). Dyspnea was statistically more frequent in the ARDS group (*p* = 0.00001) while fever was more frequent in patients without ARDS (X^2^ (1, *N* = 53) = 14.018, *p* < 0.05).

**Table 2:** General and neurological symptoms and neurological examination of patients with COVID-19

	Total N (%)	With ARDS N (%)	Without ARDS N (%)	*p*-value
**General symptoms**				
Number of patients	53	28	25	
Fever	13 (24.5)	3 (10.7)	10 (40)	**.013**
Dyspnoea	25 (47.2)	20 (71.4)	5 (20)	**.00001**
Cough	1 (1.9)	0	1 (4)	.895
Diarrhea	4 (7.5)	2 (7.1)	2 (8)	.906
Myocardial infarction	2 (3.8)	1 (3.6)	1 (4)	.937
Syncope	2 (3.8)	2 (7.1)	0	.447
Bedridden	21 (39.6)	19 (67.9)	2 (8)	**<.00001**
Other^a^	9 (17)	0	9 (36)	**.001**
**Neurological symptoms**				
Hyposmia/anosmia	14 (26.4)	2 (7.1)	12 (48)	**.003**
Ageusia	8 (15.1)	1 (3.6)	7 (28)	.092
Encephalopathy	2 (3.8)	1 (3.6)	1 (4)	.934
Headache	11 (20.6)	3 (10.7)	8 (32)	.058
Vertigo/dizziness	6 (11.3)	1 (3.6)	5 (20)	**.032**
Nausea/emesis	2 (3.8)	1 (3.6)	1 (4)	.726
Neuralgia	7 (13.2)	3 (10.7)	4 (16)	.434
General muscle weakness/pain	17 (32.1)	8 (28.6)	9 (36)	.525
**Neurological examination**				
Delirium	7 (13.2)	5 (17.9)	2 (8)	.260
Impairment in orientation	6 (11.3)	1 (3.6)	5 (20)	.073
Anisocoria	9 (17)	8 (28.6)	1 (4)	.019
Ptosis	2 (3.8)	2 (7.1)	0	.274
Nystagmus	1 (1.9)	1 (3.6)	0	.528
Dysarthria	2 (3.8)	2 (7.1)	0	.274
Aphasia	4 (7.5)	3 (10.7)	1 (4)	.350
Dysphagia	1 (1.9)	1 (3.6)	0	.528
Neglect (tactile)	2 (3.8)	0	2 (8)	.218
Meningism	1 (1.9)	1 (3.6)	0	.528
Abnormal function CN III	8 (15.1)	6 (21.4)	2 (8)	.164
Abnormal eye movement	8 (15.1)	6 (21.4)	2 (8)	.164
Abnormal function CN V	1 (1.9)	1 (3.6)	0 (0)	.528
Abnormal function CN VII	4 (7.5)	3 (10.7)	1 (4)	.395
Paresis	25 (47.2)	19 (67.9)	6 (24)	.001
Abnormal fine motor skills	6 (11.3)	5 (17.9)	1 (4)	.123
Tremor	3 (5.6)	2 (7.1)	1 (4)	.633
Spasticity	2 (3.8)	0	2 (8)	.218
Abnormal deep tendon reflexes	24 (45.3)	18 (64.3)	6 (24)	.011
Babinski sign	8 (15.1)	7 (25)	1 (4)	.037
Sensory deficit	24 (45.3)	16 (57.1)	8 (32)	.034
Impairment in coordination	39 (73.6)	21 (75)	18 (72)	.525
Unsteady gait	13 (24.5)	5 (17.9)	8 (32)	.232
Cerebellar symptoms	2 (3.8)	1 (3.6)	1 (4)	.726
Bladder dysfunction	3 (5.6)	2 (7.1)	1 (4)	.089

*ARDS* Acute respiratory distress syndrome

^a^other symptoms caused by other acute diseases independent of COVID-19, e.g. gastrointestinal bleeding

Prevalent neurological symptoms were hyposmia/anosmia, ageusia, headache and general muscle pain while symptoms indicating encephalopathy (e.g. disturbances of consciousness) were less common. Table 2 also displays all neurological abnormalities that were evident during hospitalization. The most frequent observations were paresis and abnormal deep tendon reflexes, primarily due to critical illness neuropathy or myopathy (CIN/CIM) but also as a consequence of stroke and intracerebral/subarachnoid bleeding. 15.1% (*n* = 8) of all patients displayed disturbances of the oculomotor nerve and 17% (*n* = 9) had anisocoria. Pathological reflexes (Babinski sign) were predominantly seen in patients with ARDS. Other prevalent neurological symptoms were delirium, impairment of orientation, aphasia, and disturbances in cranial nerves V and VII. Regarding cognitive performance, the majority of 13 tested patients (61.5%) showed cognitive impairment with deficits primarily in executive function, attention, language and delayed recall (Table 3).

**Table 3:** Montreal Cognitive Assessment of patients with COVID-19

Patient # (age)	Executive (max. 5)	Naming (max. 3)	Attention (max. 6)	Language (max. 3)	Abstraction (max. 2)	Delayed Recall (max. 5)	Orientation (max. 6)	Total Points (max. 30)
1 (51)	5	3	6	2	2	5	6	29
3 (32)	3	3	6	3	2	5	6	28
4 (49)	5	3	6	3	2	1	4	24
6 (77)^a^	0	3	1	1	0	0	6	11
7 (80)	4	3	4	0	1	2	6	20
11 (70)	3	3	6	2	2	2	6	24
12 (66)	4	3	6	3	2	2	6	26
14 (54)	5	3	6	3	2	2	6	27
15 (61)	5	3	4	2	2	4	6	26
18 (60)^b^	2	3	3	2	2	1	3	16
19 (59)	3	3	4	2	2	4	6	22
20 (53)	5	3	5	1	2	0	6	22
21 (75)	3	3	5	0	2	5	6	24
Mean (SD)	3.62 (1.50)	3 (0)	4.77 (1.54)	1.85 (1.07)	1.77 (0.60)	2.54 (1.85)	5.62 (0.96)	23 (5.02)

*MoCA* Montreal Cognitive Assessment. The MoCA total score corresponds to the total uncorrected score

^a^patient died during the course of the disease, ^b^patient with acute respiratory distress syndrome

With respect to further neurological complications, three patients with ARDS suffered an ischemic stroke, eight patients presented with subarachnoid bleeding and two developed seizures. Of 13 patients with CIN/CIM, eleven suffered from ARDS. Overall, ten (19%) out of 53 COVID-19 patients died, nine of the ten deceased patients were in the ARDS-group (Additional file 1).

### Blood and CSF findings

All patients received detailed laboratory testing including blood count, inflammatory values, coagulation profile, liver function tests, and kidney values. In nine patients, additional extensive inflammatory laboratory workup and lumbar puncture were performed (Table 4). Three patients had a mild pleocytosis, as well as a moderate lactate increase and disturbance of the BBB, suggesting an inflammatory CSF constellation. In these patients, D-dimers, Ferritin, Interleukin (IL)-2-receptor, Interleukin (IL)-6 and TNF-alpha levels were all increased as an expression of a general distinct inflammatory response serologically as well as intrathecally. By contrast, two patients exhibited a prominent elevation of the above-mentioned inflammatory mediators in serum without abnormalities in CSF. No SARS-CoV-2 RNA was detected in any of the CSF samples. Ten patients with ARDS showed reactivation of Varicella-zoster virus, Herpes simplex virus, Epstein-Barr virus or Cytomegalovirus (detected by serum PCR) as a sign of general immune impairment during the acute phase of COVID-19.

**Table 4:** CSF and serum analyses of patients with COVID-19

CSF parameters							
Patient # of total 53 patients (see Additional file 1 (age)	Cells/μl (< 5)	Total Protein g/l (0.2–0.4)	Lactate mmol/l (1.1–2.4)	Glucose mg/dl (40–70)	Blood-brain-barrier dysfunction	Oligoclonal Bands (Isoelectric focussing)	SARS-CoV-2 PCR
2 (62)^a^	12	0.61	2.1	2	yes	negative	negative
20 (53)	3	0.42	2.0	85	no	negative	not available
21 (75)	1	0.18	1.4	41	no	negative	negative
23 (76)	2	0.34	1.7	55	no	negative	negative
32 (69)	10	0.95	3.5	87	yes	negative	negative
33 (59)	12	0.3	4.3	83	yes	negative	negative
37 (56)	2	0.39	2.2	80	no	negative	negative
45 (77)	2	0.31	1.5	64	no	negative	negative
53 (55)^a^	1	0.19	2.8	105	no	negative	negative
Specific serum analysis							
Patient # (age)	D-dimers ng/ml (< 500)	Ferritin ng/ml (15–150)	Il-2-Rc U/ml (158–623)	Il-6 pg/ml (< 7.0)	TNF-alpha pg/ml (< 8.1)	NSE ng/ml (< 17)	
2 (62)^a^	13,662	1497	6455	4203	74.1	26	
20 (53)	292	232	452	22	10	n.a.	
21 (75)	1209	234	n.a.	n.a.	n.a.	n.a.	
23 (76)	613	415	< 158	21	6.8	n.a.	
32 (69)	23,237	2277	943	1407	25	29	
33 (59)	25,243	6644	4343	331	44	31	
37 (56)	95,052	3501	2245	1408	27	51	
45 (77)	1170	1790	1007	1369	< 8	n.a.	
53 (55)^a^	24,754	5264	n.a.	636	n.a.	n.a.	

*CSF* Cerebrospinal fluid, *Il-2-Rc* Interleukin-2 receptor, *Il-6* Interleukin-6, *TNF-alpha* Tumor necrosis factor, *NSE* Neuron-specific enolase, *n.a.* Not available

^a^patient died during the course of the disease

### Imaging findings

**Fig. 1:**
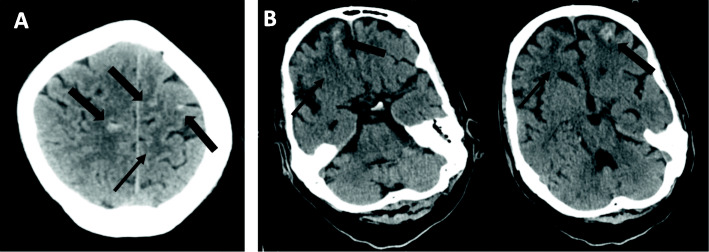
Exemplary cranial CT scans with diffuse sulcal/subarachnoidal bleedings frontally, frontobasally and parietally (wide arrows), and partly confluent frontobasal and subcortical hypodensities (slim arrows) indicating infarction in **a** a 48 year-old male (patient #39) and **b** a 56 year-old male (patient #37) with both severe COVID-19 and acute respiratory distress syndrome

**Fig. 2:**
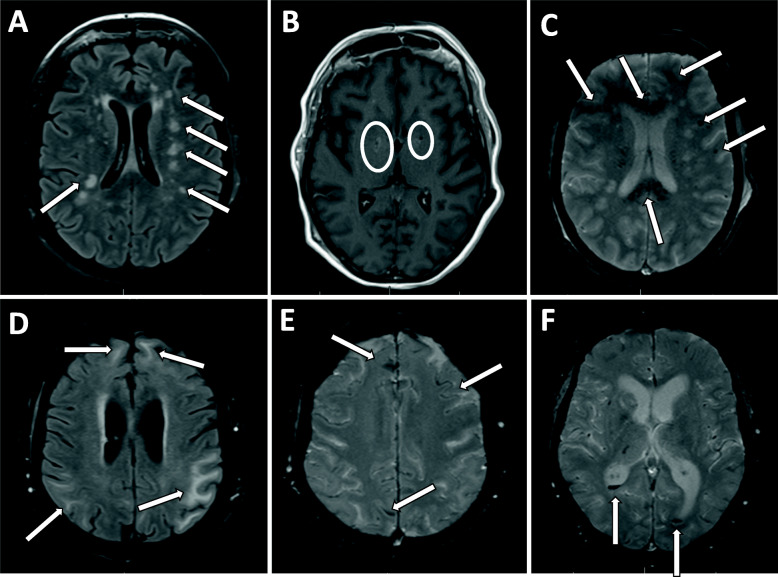
Exemplary MR imaging with multiple spotted (**a**) or confluent (**b**) hyperdense lesions (arrow) on axial fluid-attenuated inversion recovery (FLAIR) partly with contrast enhancement (circle) in the basal ganglia (**b**). Furthermore, evidence of multiple microbleeds in T2* sequences with emphasis on the corpus callosum (**c**) and the white matter as well as multiple cortical sulcal hemorrhages (**e**) and proof of ventricular hemorrhage (**f**). **a**-**c** 62 year-old female (patient #2) and **d**-**f**: 69 year-old male (patient #32), both with severe COVID-19 and acute respiratory distress syndrome

We performed CT and MR imaging in 27 patients (of those 20 with ARDS and seven without ARDS) as diagnostic work-up of patients with neurological symptoms. Twenty three of twenty seven patients were examined by CT scan, out of which five patients were additionally examined by MR imaging, and four patients received primary MR imaging without CT scan. In eight patients (29.6%), CT scan revealed subarachnoid hemorrhage in multiple brain regions, predominantly frontal and parietal cortical (Fig. 1a). Multifocal supratentorial punctual or confluent hypodense white matter lesions indicating cerebral microangiopathy or subacute ischemic lesions were detected in twelve patients (44.4%) (Fig. 1b). Both findings are considered signs of CNS damage in severely affected COVID-19 patients, most of them suffering from ARDS. Less prevalent CT findings included global cerebral atrophy (14.8%) and other incidental findings such as meningioma (one patient), a tumor suspicious lesion (one patient) and a lesion suggesting cerebral abscess or metastasis (one patient), all of them most likely not associated with COVID-19. The most frequent MRI findings reflected those of the CT with multiple bilaterally non-confluent and confluent hyperintense white matter lesions detected in fluid-attenuated inversion recovery (Fig. 2a and d) with partly contrast enhancement (Fig. 2b) as well as multiple sulcal and subarachnoid microbleeds on gradient-echo T2 sequences, sometimes involving the ventricular system (Fig. 2c, e, f). Overall, three out of 27 (11.1%) patients were diagnosed with ischemic stroke, out of those two with ARDS and one without ARDS.

### EEG findings

Evaluation of EEG of eight patients (six with ARDS, two without ARDS) showed diffuse pathological slowing and intermittent rhythmic delta-activity in four patients (Additional File 3A) and sporadic epileptic abnormalities bifrontal and bitemporal in one patient (Additional File 3B). Three patients had a normal EEG and one patient displayed sporadic epileptic abnormalities without diffuse slowing.

## Discussion

In the present study, we systematically investigated neurological deficits in COVID-19 patients. While numerous publications are available on clinical manifestations of SARS-CoV-2 infections by now, some of them focusing on neurological aspects mostly in the form of case reports or small case series, no systematic neurological investigation of in-house patients on both normal wards and intensive care units are available from Germany so far.

COVID-19 patients hospitalized at the RWTH Aachen University Hospital during the first wave of the pandemic (March through September 2020) presented mainly with the following neurological features: Coordination deficits (74%), cognitive impairment (61.5%), paresis (47%), abnormal reflex status (45%), sensory abnormalities (45%), general muscle weakness and pain (32%), hyposmia (26%), and headache (21%). Among them, patients with ARDS were neurologically more severely affected than non-ADRS patients.

Whereas 29.6% of our patients presented with sulcal bleedings, only 11.1% of our patients showed ischemic stroke associated with SARS-CoV-2 infection. Numbers of COVID-19-associated cerebrovascular events vary in the literature, with a single to high number of cases reported by different observational studies, e.g. 5% ischemic stroke in Wuhan, China [13], 2% in Milan, Italy [14], and in the Netherlands [15], while other colleagues from Brescia in Italy reported 77% [16] or 45% in the U.K. [17]. The etiology of the strokes remains unclear. While some researchers suggested a direct effect of the viral infection by either positive SARS-CoV-2 PCR in CSF or specific intrathecal antibody synthesis [2], many of the stroke-COVID-19 patients already had traditional cardiovascular risk factors. Since those patients with pre-existing cardiovascular risk factors and disease have a higher risk not only for stroke but also for a severe course of COVID-19, it is difficult to pinpoint the actual stroke etiology. What complicates things is the fact that many ICU patients suffer from disseminated intravascular coagulation [18], what further increases the risk of thromboembolic pathologies. Elevated D-dimer, fibrinogen, and the presence of antiphospholipid antibodies appear to be prominent in COVID-19 patients with concomitant acute ischemic stroke [19]. Most likely, stroke is rather correlated with COVID-19 and its hematological complications than causally are linked to the viral replication itself.

In contrast to others [2], in our cohort we did not observe a single case of SARS-CoV-2-related meningitis [6] nor acute disseminated encephalomyelitis myelitis [20–22], neither did we observe Guillain-Barré syndrome [23, 24]. Roughly 24% of our patients were diagnosed with critical illness neuro−/myopathy (CIN/CIM), a percentage that is similar to neurological ICU patients treated for other indications. Interestingly, 26% patients reported hyposmia, which is generally in line with previous reports of about 20% hyposmia in COVID-19 patients [25], emphasizing the neuro-invasive potential of SARS-CoV-2. Our EEG findings were rather unspecific. Pathological EEG findings were reported in form of epileptiform discharges and frontal sharp waves in COVID-19 patients with impaired consciousness or suspected clinical seizures [26] and most likely reflect diffuse encephalopathy.

Among the nine patients with lumbar puncture, none was positive for SARS-CoV-2 RNA in the CSF. There are single reports of positive RT-PCR results from CSF [27, 28], whereas in the majority of cases the PCR remains negative in spite of the patient’s neurological symptoms, even in cases of encephalitis [29–36]. There are various hypothesis about the viral invasion ways into the CNS, such as hematogenous spread by introduction of the virus via infected peripheral leukocytes, ACE2-mediated transmission through endothelial cells, or trans-synaptic spread into the brain via the olfactory epithelium in the nasal cavity [5]. The latter seems to be likely due to the spatial proximity of the CNS to the pharynx with high viral load. An alternative way is hematogenous spread via virus-associated damage to the BBB. In most patients with e.g. bacterial meningitis, the pathogen reaches the brain via hematogenous ways and not by propagated infection of the throat or ear, which makes hematogenous spreading of SARS-CoV2 into the CNS a possibility.

In ten patients (18.9%) the cranial nerve was affected with SARS-CoV-2, symptoms including oculomotor and/or facial nerve palsy and/or trigeminal nerve deficits. Out of these ten patients, three showed multiple cranial nerve involvement. Of the ten patients with cranial nerve involvement only two received a lumbar puncture for CSF analysis, which revealed mild pleocytosis and elevated protein concentrations in both cases (Table 4, cases #2 and #32). In both cases we detected reactivation of other viruses, such as Epstein-Barr-virus and Herpes-virus. One patient was treated with Ganciclovir, the other with Aciclovir. In summary, the diagnosis of neuritis cranialis is likely, because (i) SARS-CoV-2 PCR from CSF was negative in all our cases with CSF analysis, and (ii) total CSF protein was increased in these two cases with cranial nerve involvement but not in the majority of the other cases without cranial nerve involvement (Table 4).

In general, cerebral complications are common in patients with veno-arterial extracorporeal membrane oxygenation (ECMO) independent of Covid-19. ECMO therapy is associated with an increased risk for ischemic lesions as well as hemorrhagic lesions. In our cohort, four of the eight patients with proven subarachnoid hemorrhage were treated with ECMO. All patients in our cohort with subarachnoid hemorrhage received anticoagulation for therapeutic purpose. For instance, one patient who was not treated with ECMO had a myocardial infarction, another had thrombosis of the subclavian and jugular veins. Since the subarachnoid hemorrhages were not typical of aneurysm hemorrhage and we considered other possible causes more likely, no angiography was performed.

In our cohort, the number of patients with cognitive impairment was astonishingly high with 61.5% and must be interpreted with caution since these observations were acquired during the acute phase of COVID-19. MoCA is a good bedside test to detect cognitive decline but can yield aberrant results when applied to patients with dementia, neurodegenerative diseases of different origin, or in patients with septic encephalopathy. The degree of cognitive pathology in our cohort is high in comparison to roughly 6% (10/153) of patients presenting with “dementia-like” syndrome in the UK [17]. One of the cognitively impaired patients had a pre-existing diagnosis of a drug-treated idiopathic Parkinson’s disease, but in the other patients no pre-existing cognitive impairments were known. The other pre-existing medical conditions included arterial hypertension, diabetes mellitus to hepatitis C, polysubstance use, non-small-cell bronchial carcinoma, and metastatic cholangiocellular carcinoma.

Since the cognitive impairment of our patients during the acute phase of COVID-19 constitutes only a snapshot of the situation, further longitudinal assessment is needed to determine potential long-term complications and co-morbidities. Our data are not without a selection bias. We were only able to neurologically characterize 53 of 138 patients in depth (38.4%), although we tried to neurologically examine all patients beginning end of March 2020, regardless of the severity of the Covid-19 disease. Because patients were primarily in internal medicine wards rather than neurology wards, we missed out on some patients who, for example, died shortly after admission. The initial assumption that Covid-19 was only a pulmonary disease, was also partly responsible for the loss of patients at the beginning of the pandemic. However, because we examined all patients regardless of the severity of the disease, our report reflects the distribution of neurological symptoms whose disease stage required admission to a university hospital. Taken together, neurological abnormalities in the COVID-19 patient cohort assessed in our study were not uncommon, yet not very specific. The neurological complications of SARS-CoV2 are similar to those of other coronavirus’ diseases such as SARS and MERS [37]. It is not surprising that the most severely affected patients with ARDS or on ICU had more neurological deficits and complications than the non-ARDS patients. Some of the observed neurological symptoms are commonly found in the context of severe infectious diseases and after ICU interventions in patients with e.g. septic encephalopathy, and are therefore no unique features of COVID-19. On the other hand, other symptoms such as hyposmia, which was common in our patients like in various other studies [38–43], seem to be comparably SARS-CoV2-specific and may indeed represent one possible pathomechanism of the virus penetration into the CNS [5]. The quite common occurrence of hemorrhage with a predilection for the corpus callosum can be confirmed in out cohort. Microbleeds of the splenium are seen following traumatic head injury or in critical ill patients with a need for mechanical ventilation [44] and are - like in our patients - associated with leukencephalopathy. Both findings were predominant in male patients who showed stronger severity of symptoms and who had been hospitalized for a longer time, with worse functional outcome at discharge, or even death [45]. We can confirm this clientele in our cohort with abnormal MRI findings in patients with ARDS and/or prolonged ventilation.

In the majority of data it remains difficult to identify specific (e.g. neurotropism of SARS-CoV2, possible intrathecal viral replication) and non-specific neurological features (e.g. hypoxia, septic encephalopathy, CIN/CIM) of a disease primarily affecting the respiratory tract. Taken together, we interpret our neurological observations as mostly associated with hypoxia and septic states induced by a strongly pro-inflammatory viral infection and to a far lesser extend caused by direct viral neuronal damage.

## Conclusions

We are aware of the limitations regarding the interpretation of cognitive deficits found in this cohort, particularly in the context of the acute illness and especially in febrile or delirious patients. We therefore interpret these data rather cautiously, also because the premorbid cognitive state of the patients is unknown. Nevertheless, we assume that previously cognitively impaired patients can experience a clear and permanent aggravation in cognition after acute illnesses and that even previously cognitively non-impaired patients can manifest with dementia after severe infectious diseases associated with sepsis or delirium [46]. Since our data is limited in the context of acute events, a follow-up after 3 and 9 months, respectively, including neuropsychological testing and MRI is currently underway to detect possible permanent sequelae after COVID-19, allowing us to assess possible long-term deficits.

## Supplementary Information

**Additional file 1.** Chronological list of all investigated patients with COVID-19. List of all investigated patients including patient numbers, age, sex, specification in ARDS and non-ARDS and investigations (CSF, cranial CT or MRI, EEG) carried out in each case.

**Additional file 2.** EEG-findings. Descriptions of two pathological EEG-findings of two different patients.

**Additional file 3.** EEG-excerpts. Exemplary two excerpts of EEG-examinations of two different patients.

## Data Availability

The datasets used and/or analyzed during the current study are available from the corresponding author on reasonable request.

## Abbreviations

ACE2: Angiotensin-converting enzyme 2
ADC: Apparent diffusion coefficient
ARDS: Acute respiratory distress syndrome
CCB: Calcium channel blockers
CNS: Central nervous system
BBB: blood-brain-barrier
CIN/CIM: Critical illness neuropathy or myopathy
CT: Computed tomography
COVID-19: Corona virus disease 2019
CSF: Cerebrospinal fluid
CVVHD: Continuous veno-venous hemodialysis
Cz: Central
DWI: Diffusion-weighted imaging
ECMO: Extracorporeal membrane oxygenation
EEG: Electroencephalography
F3/F4: Frontal
FiO2: Fraction of inspired oxygen
FLAIR: Fluid-attenuated inversion recovery
Fp1/Fp2: Pre-frontal
ICU: Intensive care unit
GCS: Glasgow coma scale
Il-2: Interleukin-2
Il-6: Interleukin-6
IQR: Interquartile range
MERS: Middle east respiratory syndrome
MoCA: Montreal cognitive assessment test
MRI: Magnetic resonance imaging
NSE: Neuron specific enolase
O1/O2: Occipital
P3/P4: Parietal
PaO2: Arterial partial pressure of oxygen
PNS: Peripheral nervous system
RNA: Ribonucleic acid
RT-PCR: Real-time reverse-transcriptase polymerase chain reaction
SARS-CoV-2: Severe acute respiratory syndrome coronavirus 2
T1-T6: Temporal
TNF-alpha: Tumor necrosis factor alpha
TOF: Time-of-flight-angiography

## References

[1] Mao L, Jin H, Wang M, Hu Y, Chen S, He Q (2020). Neurologic manifestations of hospitalized patients with coronavirus disease 2019 in Wuhan, China. JAMA Neurology.

[2] Ellul MA, Benjamin L, Singh B, Lant S, Michael BD, Easton A (2020). Neurological associations of COVID-19. The Lancet Neurology.

[3] Brundin P, Nath A, Beckham JD (2020). Is COVID-19 a perfect storm for Parkinson's disease?. Trends in Neurosciences.

[4] Pavel A, Murray DK, Stoessl AJ (2020). COVID-19 and selective vulnerability to Parkinson’s disease. The Lancet Neurology.

[5] Desforges M, Le Coupanec A, Brison E, Meessen-Pinard M, Talbot PJ (2014). Neuroinvasive and neurotropic human respiratory coronaviruses: Potential neurovirulent agents in humans. Advances in Experimental Medicine and Biology.

[6] Desforges M, Le Coupanec A, Dubeau P, Bourgouin A, Lajoie L, Dubé M, Talbot PJ (2019). Human coronaviruses and other respiratory viruses: Underestimated opportunistic pathogens of the central nervous system?. Viruses.

[7] Xu H, Zhong L, Deng J, Peng J, Dan H, Zeng X (2020). High expression of ACE2 receptor of 2019-nCoV on the epithelial cells of oral mucosa. International Journal of Oral Science.

[8] van Riel D, Verdijk R, Kuiken T (2015). The olfactory nerve: A shortcut for influenza and other viral diseases into the central nervous system. Journal of Pathology.

[9] Förster, M., Weyers, V., Küry, P., Barnett, M., Hartung, H.-P., & Kremer, D. (2020). Neurological manifestations of SARS-CoV-2 - a controversy “gone viral”. *Brain Communications.*10.1093/braincomms/fcaa149. 10.1093/braincomms/fcaa149 PMC7543269 33210085

[10] Balfanz P, Hartmann B, Müller-Wieland D, Kleines M, Häckl D, Kossack N (2021). Early risk markers for severe clinical course and fatal outcome in German patients with COVID-19. PLoS One.

[11] Ranieri VM, Rubenfeld GD, Thompson BT, Ferguson ND, Caldwell E, ARDS Definition Task Force (2012). Acute respiratory distress syndrome: The Berlin definition. JAMA.

[12] Nassredine ZS, Phillips NA, Bédirian V, Charbonneau S, Whitehead V, Collin I (2005). Montreal cognitive assessment, MoCA: A brief screening tool for mild cognitive impairment. Journal of the American Geriatrics Society.

[13] Li Y, Li M, Wang M, Zhou Y, Chang J, Xian Y (2020). Acute cerebrovascular disease following COVID-19: A single center, retrospective, observational study. Stroke and Vascular Neurology.

[14] Lodigiani C, Iapichino G, Carenzo L, Cecconi M, Ferrazzi P, Sebastian T (2020). Venous and arterial thromboembolic complications in COVID-19 patients admitted to an academic hospital in Milan, Italy. Thrombosis Research.

[15] Klok FA, Kruip MJHA, van der Meer NJM, Arbous MS, Gommers DAMPJ, Kant KM (2020). Incidence of thrombotic complications in critically ill ICU patients with COVID-19. Thrombosis Research.

[16] Benussi A, Pilotto A, Premi E, Libri I, Giunta M, Agosti C (2020). Clinical characteristics and outcomes of inpatients with neurologic disease and COVID-19 in Brescia, Lombardy, Italy. Neurology.

[17] Varatharaj A, Thomas N, Ellul MA, Davies NWS, Pollak TA, Tenorio EL (2020). Neurological and neuropsychiatric complications of COVID-19 in 153 patients: A UK-wide surveillance study. The Lancet Psychiatry.

[18] Wijdicks EF, Silbert PL, Jack CR, Parisi JE (1994). Subcortical hemorrhage in disseminated intravascular coagulation associated with sepsis. American Journal of Neuroradiology.

[19] Tan Y-K, Goh C, Leow AST, Tambyah PA, Ang A, Yap E-S (2020). COVID-19 and ischemic stroke: A systematic review and meta-summary of the literature. Journal of Thrombosis and Thrombolysis.

[20] Moriguchi T, Harii N, Goto J, Harada D, Sugawara H, Takamino J (2020). A first case of meningitis/encephalitis associated with SARS-Coronavirus-2. International Journal of Infectious Diseases.

[21] Zanin L, Saraceno G, Panciani PP, Renisi G, Signorini L, Migliorati K, Fontanella MM (2020). SARS-CoV-2 can induce brain and spine demyelinating lesions. Acta Neurochirurgica.

[22] Zhang, T., Rodricks, M. B., & Hirsh, E. (2020). COVID-19-associated acute disseminated encephalomyelitis: A case report. *medRxiv*. 10.1101/2020.04.16.20068148.

[23] Reichard RR, Kashani KB, Boire NA, Constantopoulos E, Guo Y, Lucchinetti CF (2020). Neuropathology of COVID-19: A spectrum of vascular and acute disseminated encephalomyelitis (ADEM)-like pathology. Acta Neuropathologica.

[24] Toscano G, Palmerini F, Ravaglia S, Ruiz L, Invernizzi P, Cuzzoni MG (2020). Guillain–Barré syndrome associated with SARS-CoV-2. The New England Journal of Medicine.

[25] Bénézit F, Le Turnier P, Declerck C, Paillé C, Revest M, Dubée V, RAN COVID Study Group (2020). Utility of hyposmia and hypogeusia for the diagnosis of COVID-19. The Lancet Infectious Diseases.

[26] Galanopoulou AS, Ferastraoaru V, Correa DJ, Cherian K, Duberstein S, Gursky J (2020). EEG findings in acutely ill patients investigated for SARS-CoV-2/COVID-19: A small case series preliminary report. Epilepsia Open.

[27] Lersy, F., Benotmane, I., Helms, J., Collange, O., Schenck, M., Brisset, J.-C., … Kremer, S. (2020). Cerebrospinal fluid features in COVID-19 patients with neurological manifestations: Correlation with brain MRI findings in 58 patients. *The Journal of Infectious Diseases.*10.1093/infdis/jiaa745. 10.1093/infdis/jiaa745 PMC7798956 33249438

[28] Lewis, A., Frontera, J., Placantonakis, D. G., Lighter, J., Galetta, S., Balcer, L., & Melmed, K. (2021). Cerebrospinal fluid in COVID-19: A systematic review of the literature. *Journal of the Neurological Sciences*. 10.1016/j.jns.2021.117316. 10.1016/j.jns.2021.117316 PMC7833669 33561753

[29] Li YC, Bai WZ, Hashikawa T (2020). The neuroinvasive potential of SARS-CoV2 may play a role in the respiratory failure of COVID-19 patients. Journal of Medical Virology.

[30] Zhou P, Yang X-L, Wang X-G, Hu B, Zhang L, Si H-R (2020). A pneumonia outbreak associated with a new coronavirus of probable bat origin. Nature..

[31] Neumann B, Schmidbauer ML, Dimitriadis K, Otto S, Knier B, Niesen W-D (2020). Cerebrospinal fluid findings in COVID-19 patients with neurological symptoms. Journal of the Neurological Sciences.

[32] Frontera JA, Sabadia S, Lalchan R, Fang T, Flusty B, Millar-Vernetti P (2020). A prospective study of neurologic disorders in hospitalized COVID-19 patients in new York City. Neurology.

[33] Paterson RW, Brown RL, Benjamin L, Nortley R, Wiethoff S, Bharucha T (2020). The emerging spectrum of COVID-19 neurology: Clinical, radiological and laboratory findings. Brain.

[34] Placantonakis DG, Aguero-Rosenfeld M, Flaifel A, Colavito J, Inglima K, Zagzag D (2020). SARS-CoV-2 is not detected in the cerebrospinal fluid of Encephalopathic COVID-19 patients. Frontiers in Neurology.

[35] Heming M, Li X, Räuber S, Mausberg AK, Börsch A-L, Hartlehnert M (2021). Neurological manifestations of COVID-19 feature T cell exhaustion and dedifferentiated monocytes in cerebrospinal fluid. Immunity.

[36] Al Saiegh F, Ghosh R, Leibold A, Avery MB, Schmidt RF, Theofanis T (2020). Status of SARS-CoV-2 in cerebrospinal fluid of patients with COVID-19 and stroke. Journal of Neurology, Neurosurgery & Psychiatry.

[37] Arabi YM, Harthi A, Hussein J, Bouchama A, Johani S, Hajeer AH (2015). Severe neurologic syndrome associated with Middle East respiratory syndrome corona virus (MERS-CoV). Infection.

[38] Giacomelli A, Pezzati L, Conti F, Bernacchia D, Siano M, Oreni L (2020). Self- reported olfactory and taste disorders in SARS-CoV-2 patients: A cross-sectional study. Clinical Infectious Diseases.

[39] Xydakis MS, Dehgani-Mobarski P, Holbrook EH, Geisthoff UW, Bauer C, Hautefort C (2020). Smell and taste dysfunction in patients with COVID-19. The Lancet Infectious Diseases.

[40] Hopkins, C. & Kumar, N. (2020). Loss of sense of smell as marker of COVID-19 infection. Resource document. https://www.entuk.org/loss-sense-smell-marker-covid-19-infection Accessed 4 June 2020

[41] Hopkins C, Surda P, Kumar N (2020). Presentation of new onset anosmia during the COVID-19 pandemic. Rhinology.

[42] Butowt R, Bilinska K (2020). SARS-CoV-2: Olfaction, brain infection, and the urgent need for clinical samples allowing earlier virus detection. ACS Chemical Neuroscience.

[43] Cooper KW, Brann DH, Farruggia MC, Bhutani S, Pellegrino R, Tsukahara T (2020). COVID-19 and the chemical senses: Supporting players take center stage. Neuron.

[44] Dixon L, McNamara C, Gaur P, Mallon D, Coughlan C, Tona F (2020). Cerebral microhaemorrhage in COVID-19: A critical illness related phenomenon?. Stroke and Vascular Neurology.

[45] Agarwal S, Jain R, Dogra S, Krieger S, Lewis A, Nguyen V (2020). Cerebral microbleeds and Leukoencephalopathy in critically ill patients with COVID-19. Stroke.

[46] Tauber, S. C., Djukic, M., Gossner, J., Eiffert, H., Brück, W., & Nau, R. (2020). Sepsis-associated encephalopathy and septic encephalitis: An update. *Expert Review of Anti-Infective Therapy*. 10.1080/14787210.2020.1812384. 10.1080/14787210.2020.1812384 32808580

