# Supplementary material for: Neurological symptoms in COVID-19: a cross-sectional monocentric study of hospitalized patients
Source: Neurol Res Pract. 2021 Mar 12;3:17. doi: 10.1186/s42466-021-00116-1 (PMC7953515; doi:10.1186/s42466-021-00116-1)
Supplement: Supplementary file 1 — Additional file 1. Chronological list of all investigated patients with COVID-19. List of all investigated patients including patient numbers, age, sex, specification in ARDS and non-ARDS and investigations (CSF, cranial CT or MRI, EEG) carried out in each case. [file 42466_2021_116_MOESM1_ESM.docx]

**Additional file 1:** Chronological list of all investigated patients with COVID-19

| Patient #  (in chronological order) | Age  (years) | Sex  (0=male, 1= female) | Evidence of ADRS | CSF | MoCA | EEG | CT | MRI |
| --- | --- | --- | --- | --- | --- | --- | --- | --- |
| 1 | 51 | 0 |  |  | x |  |  |  |
| 2* | 62 | 1 | x | x |  | x | x | x |
| 3 | 32 | 1 |  |  | x |  |  |  |
| 4 | 49 | 0 |  |  | x |  |  |  |
| 5 | 85 | 1 |  |  |  |  |  |  |
| 6 | 77 | 1 |  |  | x |  |  |  |
| 7* | 80 | 0 |  |  | x |  |  |  |
| 8 | 68 | 0 |  |  |  |  |  |  |
| 9 | 39 | 1 |  |  |  |  |  |  |
| 10 | 81 | 0 |  |  |  |  |  |  |
| 11 | 70 | 1 |  |  | x |  |  |  |
| 12 | 66 | 0 |  |  | x |  |  |  |
| 13 | 45 | 1 | x |  |  |  |  |  |
| 14 | 54 | 1 |  |  | x |  |  |  |
| 15 | 61 | 1 |  |  | x |  |  | x |
| 16 | 81 | 0 |  |  |  |  | x |  |
| 17 | 44 | 0 |  |  |  |  |  |  |
| 18 | 60 | 0 | x |  | x | x | x | x |
| 19 | 59 | 0 |  |  | x | x |  |  |
| 20 | 53 | 0 |  | x | x | x |  | x |
| 21 | 75 | 1 |  | x | x |  |  | x |
| 22 | 48 | 1 |  |  |  |  |  |  |
| 23 | 76 | 0 |  | x |  |  | x | x |
| 24 | 80 | 0 |  |  |  |  |  |  |
| 25 | 77 | 1 |  |  |  |  |  |  |
| 26 | 34 | 1 |  |  |  |  |  |  |
| 27 | 83 | 0 |  |  |  |  | x |  |
| 28 | 50 | 1 | x |  |  |  |  |  |
| 29 | 63 | 1 | x |  |  |  |  |  |
| 30 | 73 | 0 | x |  |  | x | x |  |
| 31 | 63 | 0 | x |  |  | x |  |  |
| 32 | 69 | 0 | x | x |  | x | x | x |
| 33 | 59 | 1 | x | x |  |  | x |  |
| 34 | 59 | 0 | x |  |  |  | x |  |
| 35 | 60 | 0 | x |  |  |  |  |  |
| 36* | 68 | 1 | x |  |  |  |  |  |
| 37 | 56 | 0 | x | x |  |  | x |  |
| 38 | 67 | 1 | x |  |  |  | x |  |
| 39* | 48 | 0 | x |  |  |  | x |  |
| 40* | 39 | 0 | x |  |  |  | x |  |
| 41 | 62 | 0 | x |  |  |  | x |  |
| 42* | 68 | 0 | x |  |  |  |  |  |
| 43 | 61 | 0 | x |  |  |  | x |  |
| 44 | 58 | 0 | x |  |  |  | x |  |
| 45 | 77 | 1 | x | x |  | x | x | x |
| 46 | 78 | 0 | x |  |  |  | x |  |
| 47 | 67 | 0 | x |  |  |  | x |  |
| 48* | 68 | 1 | x |  |  |  | x |  |
| 49* | 62 | 0 | x |  |  |  |  |  |
| 50* | 73 | 1 | x |  |  |  | x |  |
| 51 | 54 | 0 | x |  |  |  | x |  |
| 52 | 61 | 0 |  |  |  |  |  | x |
| 53* | 55 | 0 | x | x |  |  | x |  |

*patient died during the course of the disease
