# Supplementary material for: Neurological symptoms in COVID-19: a cross-sectional monocentric study of hospitalized patients
Source: Neurol Res Pract. 2021 Mar 12;3:17. doi: 10.1186/s42466-021-00116-1 (PMC7953515; doi:10.1186/s42466-021-00116-1)
Supplement: Supplementary file 2 — Additional file 2. EEG-findings. Descriptions of two pathological EEG-findings of two different patients. [file 42466_2021_116_MOESM2_ESM.docx]

**Additional file 2: EEG-findings:** Intermittent bilateral rhythmic Delta-activity and slowing (additional file 3A) in the electroencephalogram of a 60 year-old male (patient #18) and 63 year-old female (patient #31) with both severe COVID-19 and acute respiratory distress syndrome, in the latter slowing was accompanied by sporadic epileptic abnormalities bifrontal (additional file 3B).
