# Supplementary figures and images for: Neurological symptoms in COVID-19: a cross-sectional monocentric study of hospitalized patients
Source: Neurol Res Pract. 2021 Mar 12;3:17. doi: 10.1186/s42466-021-00116-1 (PMC7953515; doi:10.1186/s42466-021-00116-1)

**A**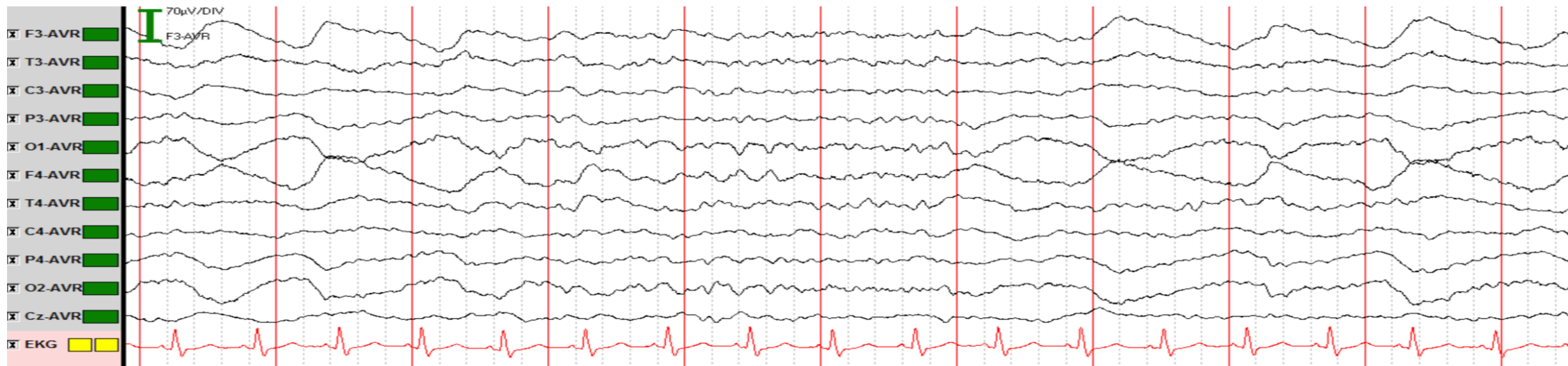**B**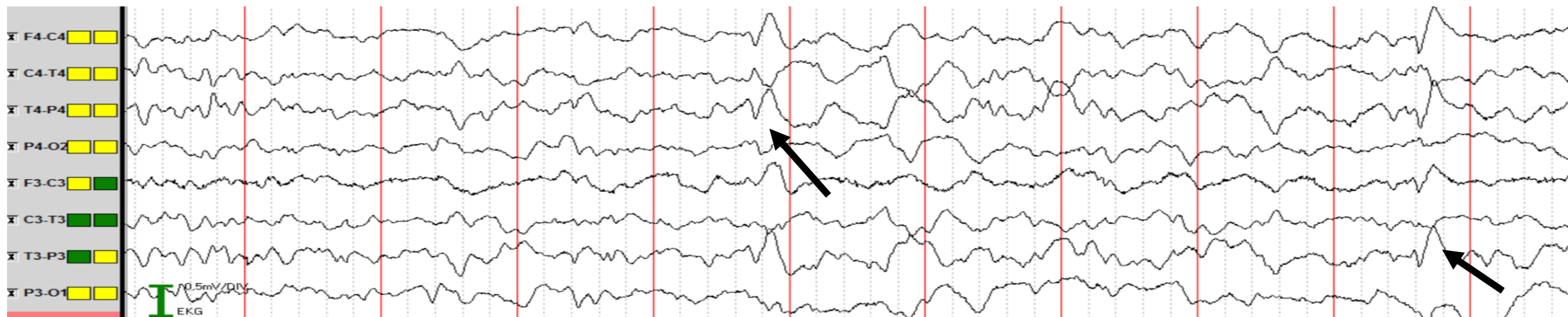

Supplement: Supplementary file 3 — Additional file 3. EEG-excerpts. Exemplary two excerpts of EEG-examinations of two different patients. [file 42466_2021_116_MOESM3_ESM.pdf]
